# Leg mechanics contribute to establishing swing phase trajectories during memory-guided stepping movements in walking cats: a computational analysis

**DOI:** 10.3389/fncom.2015.00116

**Published:** 2015-09-24

**Authors:** Keir G. Pearson, Naik Arbabzada, Rod Gramlich, Masahiro Shinya

**Affiliations:** Department of Physiology, University of AlbertaEdmonton, AB, Canada

**Keywords:** leg mechanics, obstacle avoidance, working memory, walking, model leg movement

## Abstract

When quadrupeds stop walking after stepping over a barrier with their forelegs, the memory of barrier height and location is retained for many minutes. This memory is subsequently used to guide hind leg movements over the barrier when walking is resumed. The upslope of the initial trajectory of hind leg paw movements is strongly dependent on the initial location of the paw relative to the barrier. In this study, we have attempted to determine whether mechanical factors contribute significantly in establishing the slope of the paw trajectories by creating a four-link biomechanical model of a cat hind leg and driving this model with a variety of joint-torque profiles, including average torques for a range of initial paw positions relative to the barrier. Torque profiles for individual steps were determined by an inverse dynamic analysis of leg movements in three normal cats. Our study demonstrates that limb mechanics can contribute to establishing the dependency of trajectory slope on the initial position of the paw relative to the barrier. However, an additional contribution of neuronal motor commands was indicated by the fact that the simulated slopes of paw trajectories were significantly less than the observed slopes. A neuronal contribution to the modification of paw trajectories was also revealed by our observations that both the magnitudes of knee flexor muscle EMG bursts and the initial knee flexion torques depended on initial paw position. Previous studies have shown that a shift in paw position prior to stepping over a barrier changes the paw trajectory to be appropriate for the new paw position. Our data indicate that both mechanical and neuronal factors contribute to this updating process, and that any shift in leg position during the delay period modifies the working memory of barrier location.

## Introduction

An important issue in motor physiology is the extent to which the mechanical properties of the muscular and skeletal elements of the limbs and body contribute to enhancing the neuronal command signals regulating muscle contractions. In some instances, mechanical properties are known to contribute substantially to the production of effective movements. One of the clearest examples is walking in humans. Studies with mechanical bipedal walking devices have shown that coordinated stepping can be produced without any active elements thus indicating that the mechanical properties of the legs play an important role in the production of normal bipedal locomotion (Collins et al., 2005). Our interest in this issue comes from observations on the trajectories of paw movements in cats when they step over obstacles (McVea and Pearson, 2006, 2007; McVea et al., 2009; Lajoie et al., 2010; Pearson and Gramlich, 2010). When cats begin walking after stepping over a barrier with their forelegs, the slopes of the trajectories of the hind leg paws as they move toward the barrier is highly dependent on the initial distance of the paw from the barrier (McVea and Pearson, 2006). Moreover, if the animal adjusts the position of the hind paws relative to the barrier prior to resuming walking, the trajectory slopes are appropriate for the new paw position (Pearson and Gramlich, 2010). Because these adjustments change the geometry of the hind leg prior to stepping over the barrier, the question is whether the modification of the paw trajectories might simply be due to alterations in the mechanical arrangement of the hind legs. Alternatively, a combination of altered neuronal commands and altered leg geometry could underlie the modified stepping movements. It is well known that activity in leg flexor muscles is highly modulated when cats step over obstacles (Drew, 1993; Widajewicz et al., 1994), and that the pattern of modulation of foreleg flexors depends on the size and width of the obstacles (Drew, 1993; Krouchev and Drew, 2013). However, the extent to which hind leg flexor activity is dependent on the initial paw location relative to the *same* obstacle has not been examined in previous studies, so the relative contributions of neuronal and mechanical factors in establishing the slopes of the hind paw trajectories is unknown. It is also conceivable that the relative importance of mechanical and neuronal factors differs significantly during uninterrupted stepping over an obstacle (previous studies) compared to stepping over a remembered obstacle from a standing start (this study).

The importance of distinguishing between these two possibilities is that if the former is true it would have implications for our understanding of the long-lasting working memory system involved in the production of hind leg movements to clear the remembered barrier (McVea and Pearson, 2006). In particular, it would indicate that the change in the slope of the paw trajectory associated with a change in geometry of the leg before stepping (Pearson and Gramlich, 2010) does not depend on updating information representing paw position in the working memory system. Instead the same commands to hind leg flexor muscles, established by a representation in working memory of only the required height of the step to avoid the barrier, could produce different paw trajectories simply because of differences in leg mechanics associated with differences in the initial geometry of the leg.

To gain insight into the extent to which mechanical factors contribute to the dependence of paw trajectories on initial paw position, we have developed a four-link mechanical model of a cat hind leg and examined paw trajectories in response to a variety of joint-torque profiles. First, we demonstrate that the model accurately reproduces the profiles of paw trajectories when driven by hip, knee, ankle, and paw torques derived from an inverse dynamic analysis of a hind leg when it steps over an obstacle from many different initial positions. This demonstrated that the numerical integrations necessary for the forward dynamic simulations were adequate. Next we used the profiles of average joint torques for a small range of initial paw positions to drive the model over the full range of initial paw positions observed and compared the early slopes of the paw trajectories observed in a stepping animal with the initial slopes produced by the forward model. Our expectation was that there would be close match of these slopes if modification of paw trajectories was entirely dependent on the initial geometry of the leg. Any systematic differences in these slopes would indicate some sort of modification of neuronal commands and hence provide evidence that the working memory system not only represents information about the initial paw position relative to the barrier, but also has the capacity to be updated when the initial paw position changes prior to stepping.

## Materials and methods

The primary objective of this study was to use a biomechanical model of a cat hind leg to investigate the contribution of mechanical factors in establishing the initial trajectory of the paw when the hind leg steps over a barrier. Thus, we begin this section with a description of the model, and the procedure we used to estimate joint torques from an inverse dynamic analysis of leg movements in stepping cats. Next we describe how we used the estimated joint torques as input to the model (forward dynamic simulation) to investigate how the paw trajectories are influenced by the initial geometry of the leg independent of any variation in neuronal commands. To verify some of the conclusions from this computational analysis, we also recorded electromyograms (EMGs) from hind leg flexor muscles in three cats as they stepped over a barrier. The method for recording and analyzing EMGs is described at the end of this section.

All experimental protocols were approved the University of Alberta Health Sciences Animal Policy and Welfare Committee.

### Four-link mechanical model of the cat hind leg

**Figure 1:**
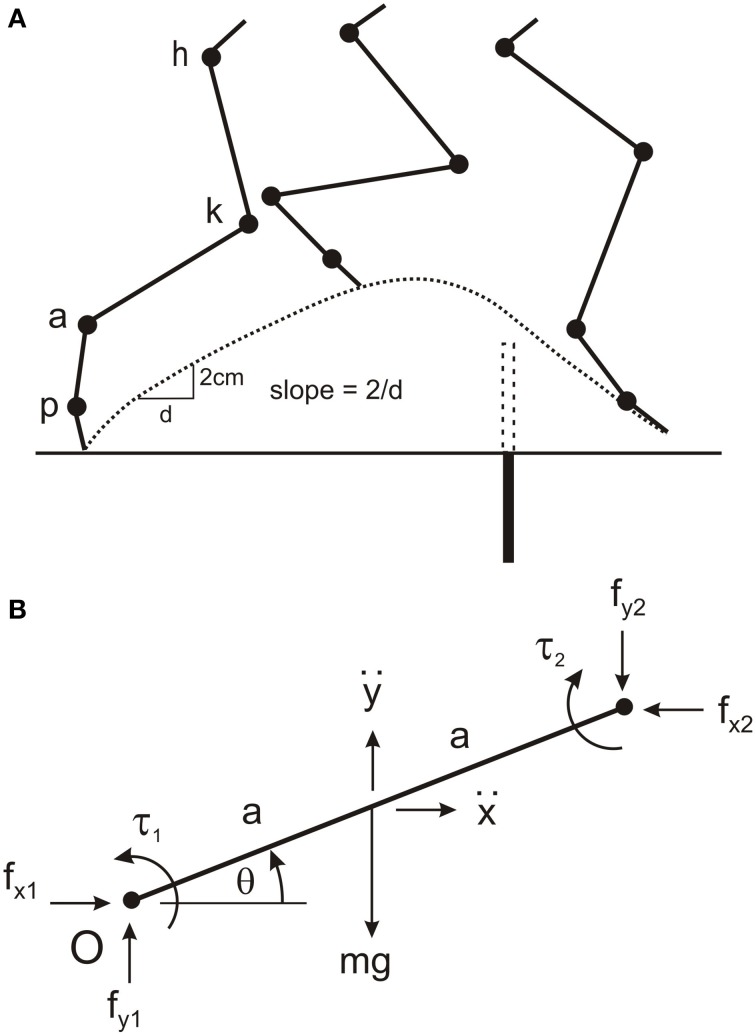
**Four-link modeling of cat hind leg**. **(A)** The cat hind leg was modeled by four uniform rods for the thigh, shank, paw, and toes connected at the hip joint (h), knee joint (k), ankle joint (a), and paw joint (p). The model was used to calculate torques at these four joints when the animal stepped over a remembered barrier (dotted rectangle), and to simulate leg movements when driven by different torque profiles. The barrier (filled rectangle) was lowered after the forelegs have stepped over it. The slope of the toe trajectory (dotted line) was calculated over distance of 2 cm soon after the swing phase commenced. **(B)** Parameters used to determine the equations of motion for a single segment (see text for details). This segment represents the first segment in a kinematic chain starting from the origin (O), which is the hip joint in the model. Note that for each segment the segment angle (θ) is measured in the anti-clockwise direction from the positive X-axis proximal to the segment. By convention, positive torques are in the anti-clockwise direction. The reaction torque and forces at the distal joint are in the opposite directions to those at the proximal joint.

The four main segments of the cat hind leg (thigh, shank, paw, and toes) were modeled as uniform rigid rods (Figure 1A). This is an enormous simplification of the complex mechanics of the leg but it allows a straightforward determination of the equations of motion and provides a simple approach for estimating torques at the hip, knee, ankle, and paw joints. A similar simplification has been used to calculate joint torques in many other biomechanical studies of hind leg movements in the cat (Hoy and Zernicke, 1985; Hoy et al., 1985; Ekeberg and Pearson, 2005). From measurements on a number of animals, the values we used for the mass of each segment were: thigh—200 gms, shank—100 gms, paw—40 gms, toe—20 gms. The lengths of the thigh and shanks were set at 10 and 11 cm, respectively, and the lengths of the paw and toes were measured from video recordings, approximately 5 and 2 cm, respectively. To get an estimate of joint torques from an inverse dynamic analysis we first derived five equations of motion for each segment (see below) thus resulting in twenty equations of motion with 20 unknowns that included the torques at the hip, knee, ankle, and paw joints. These equations were solved using custom written software in the Matlab programming language.

The independent parameters of segment angles, angular velocity, and angular acceleration required for the inverse dynamic analysis (see Equations below) were obtained from video analysis of hind leg movements in three cats stepping over a remembered barrier. Animals first stepped over a 6.5 cm high barrier with their forelegs to establish a memory of barrier height, straddled the position of the barrier for about 30 s while the barrier was lowered, and then walked forward. We have reported in previous studies (McVea and Pearson, 2006; Pearson and Gramlich, 2010) that in this situation the hind legs step high in a manner that would avoid the barrier had it remained in place. Video images were captured at 60 frames/s using the Peak Motus data acquisition system, and the positions of reflective markers on the iliac crest, hip joint, ankle joint, paw joint, and the end of the lateral toe of the right hind leg were digitized. Triangulation was used to determine knee position because of large skin movements at the knee (hence the reason for using fixed lengths of the thigh and shank). Matlab software was used to calculate joint angles for each 16.67 ms video frame (angles measured counterclockwise from right horizontal, Figure 1B), then to filter these angles (2nd order Butterworth, cutoff = 10 Hz) and interpolate joint angles between video frames to yield a time resolution of 1 ms. The angular velocities and angular accelerations of the segments were calculated by taking the 1 ms time differences in segment angles and segment angular velocities, respectively.

The positions of the end of the toes were also digitized and used to determine the slope of the toe trajectory during the elevation phase of the leg (Figure 1A), and the initial distance of the toes from the barrier. The slope of the toe trajectories were calculated by dividing the change in vertical position (2 cm) by the change in horizontal distance as the toe moved between the heights of 3 and 5 cm. Our previous studies have reported that the slope of toe trajectory is highly dependent on the initial distance from the barrier, and modified appropriately when toe position is changed prior to stepping (Pearson and Gramlich, 2010).

### Equations of motion for inverse dynamic analysis

A set of 20 equations with 20 unknowns was used to estimate joint torques. The unknowns were joint torques (four in total), horizontal and vertical joint interaction forces at each joint (eight in total), and linear horizontal and vertical accelerations at the center of mass of each segment (eight in total). For each segment there were three equations describing the dynamics and two equations describing the kinematic. The point of reference for deriving all equations was the hip joint, and the variables are illustrated for single segment in Figure 1B. The three equations for the dynamics of each segment are:

$$\begin{array}{l} \;f_{x1}-f_{x2}=m\overset{..}{x}\\ f_{y1}-f_{y2}-mg=m\overset{..}{y} \end{array}$$

$$\begin{array}{l} \tau_{1}-\tau_{2}+f_{x1}a\sin(\theta )+f_{x2}a\sin(\theta )\\ \;-f_{y1}a\cos(\theta )-f_{y2}a\cos(\theta )=I\ddot{\theta } \end{array}$$

where *g* is acceleration due to gravity, *f*_*x*1_, *f*_*y*1_and *f*_*x*2_, *f*_*y*2_are the pairs of horizontal and vertical joint interaction forces at the proximal and distal joints, respectively (note for the toe segment *f*_*x*2_ and*f*_*y*2_are zero), ẍ and $\ddot{y}$are the linear horizontal and vertical accelerations of the center of mass, respectively, τ_1_and τ_2_ are the torques at the proximal and distal joint respectively (note for the toe segment τ_2_is zero), *m* is the mass of the segment, *a* is half the length of the segment, *I* is the moment of inertia of the segment ($I=\frac{1}{3}ma^{2}$),θ is the segment angle measured anticlockwise from the horizontal right of the joint, and $\ddot{\theta }$ is the angular acceleration of the segment.

The two kinematic equations for each segment become increasingly complex going from the proximal to distal segments because of the contributions of proximal segments to the movements of the distal segments. For the most proximal segment (thigh) the two equations are:

$$\begin{array}{l} \overset{..}{x}=-a\cos(\theta ){\dot{\theta }}^{2}\;-\;a\sin(\theta )\ddot{\theta }\\ \overset{..}{y}=-a\sin(\theta ){\dot{\theta }}^{2}\;+\;a\cos(\theta )\ddot{\theta } \end{array}$$

where $\dot{\theta }$ is the angular velocity of the segment. For the most distal segment (toe) the two equations are:

(1) $$\begin{array}{l} \overset{..}{x}=-A\cos(\theta_{1}){\dot{\theta }}_{1}^{2}-A\sin(\theta_{1}){\ddot{\theta }}_{1}-B\cos(\theta_{2}){\dot{\theta }}_{2}^{2}-B\sin(\theta_{2}){\ddot{\theta }}_{2}\\ \;-C\cos(\theta_{3}){\dot{\theta }}_{3}^{2}-C\sin(\theta_{3}){\ddot{\theta }}_{3}-d\cos(\theta_{4}){\dot{\theta }}_{4}^{2}-d\sin(\theta_{4}){\ddot{\theta }}_{4} \end{array}$$

(2) $$\begin{array}{l} \overset{..}{y}=-A\sin(\theta_{1}){\dot{\theta }}_{1}^{2}+A\cos(\theta_{1}){\ddot{\theta }}_{1}-B\sin(\theta_{2}){\dot{\theta }}_{2}^{2}+B\cos(\theta_{2}){\ddot{\theta }}_{2}\\ \;-C\sin(\theta_{3}){\dot{\theta }}_{3}^{2}+C\cos(\theta_{3}){\ddot{\theta }}_{3}-d\sin(\theta_{4}){\dot{\theta }}_{4}^{2}+d\cos(\theta_{4}){\ddot{\theta }}_{4} \end{array}$$

where *A, B*, and *C* are the lengths of the thigh, shank, and paw, respectively, *d* is half the length of the toe, and the subscripts *1, 2, 3*, and *4* indicate the segment angles, angular velocities, and angular accelerations of the thigh, shank, paw, and toe, respectively. The pair of kinematic equations for the shank and paw are the first four and six terms of Equations (1) and (2), respectively, with the shank length *B* replaced by the half length *b* in the equations for the shank, and with the paw length *C* replaced by the half length *c* in the equations for the paw.

### Forward dynamic simulation of swing

The objective of using a forward dynamic simulation of the leg was to determine how a variety of torque profiles influenced the initial trajectory of the toe during swing. In particular, we wanted to examine theoretically the trajectories when the *same* torque profiles drove leg movements from *different* initial toe distances from the barrier. That is, to examine toe trajectories assuming there are no changes in neuronal commands. It is important to note that we are making the assumption that joint torques are a good proxy measure of neuronal commands, which is not necessarily accurate if the moment arms of the leg muscles varies with the initial geometry of the leg, and there are non-linear relationships between neuronal commands and muscle forces. Obviously these factors might contribute significantly to the generation of joint torques, but this cannot be assessed because little is currently known about the neuro-mechanical properties of the hind leg flexor muscles that produce the swing phase. Despite not incorporating muscle properties into our simulation, we believe using a simple four-link model of a hind leg driven by joint torques can still yield some insight into the extent that purely mechanical factors are involved in establishing the relationship between toe trajectory slope and initial toe position from the barrier.

Custom written Matlab software was used for the forward simulation. The same equations of motions, segment masses, and segment lengths used in the inverse dynamic analysis (see above) were used in the forward simulation. The fourth-order Runge-Kutta algorithm was used for numerical integration with a time step of 1 ms. Starting from the initial values of joint angles, this algorithm determined the joint angular accelerations at each time step and used these accelerations to update the joint angles and velocities for the next time step. The initial values of angular velocities required for the forward simulation were chosen to match the selection of torque profiles. For example, when using torque profiles for trials with large toe-to-barrier distance, we used the average initial angular velocities for the same subset of trials.

**Figure 2:**
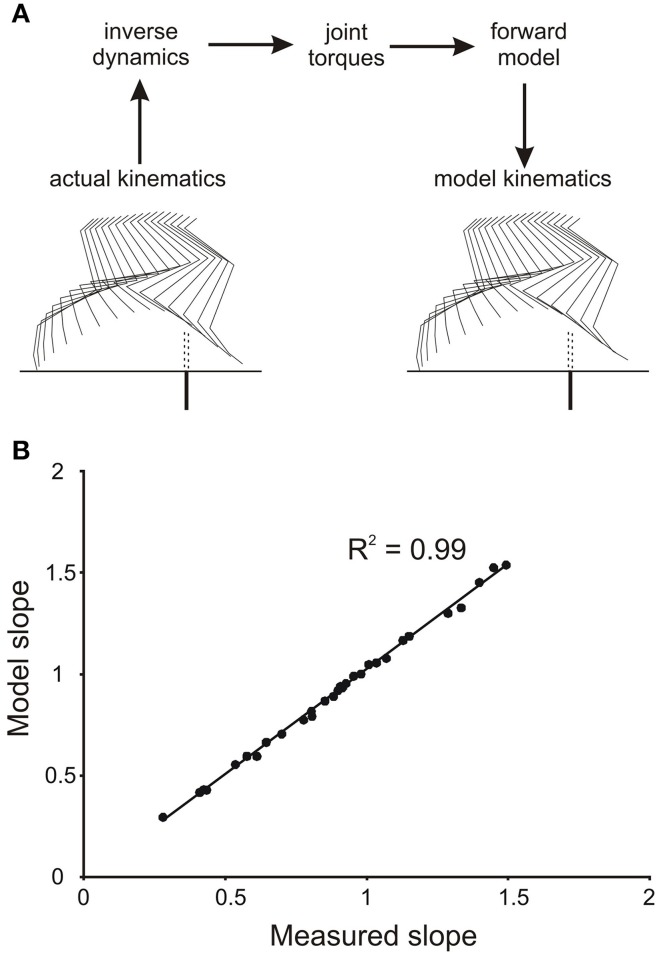
**Verification of accuracy of numerical integration of the forward mechanical model**. **(A)** The kinematics of the hind leg of a cat stepping over a barrier (actual kinematics) were used in an inverse dynamic analysis to first calculate the torque profiles at the four joints. These torque profiles were then used as inputs to the forward model to yield a simulated leg movement (model kinematics). Note the high degree of similarity of the stick figures for the actual and simulated leg movements. **(B)** Plot of the slopes of the actual and simulated toe trajectories showing a very close correspondence.

To demonstrate that the numerical integration was accurate, and that the forward simulation produced appropriate leg movements, we initially drove the forward model with the profiles of joint torques derived from the inverse dynamic analysis of single trials. Figure 2A shows sets of stick diagrams of leg geometry for a single stepping trial measured from a stepping animal (left) and generated by the forward model (right). During the early part of the swing phase the leg movements produced by the forward model closely matched the leg movements observed in the behaving animals. This was illustrated by the close correspondence of slopes of the toe trajectories in the simulation and in the behaving animal (Figure 2B). Small difference in geometry occurred near the end of the step (Figure 2A), but these occur well beyond the times of the events we were measuring (early in the swing phase). Similar matches were obtained for all trials we examined, regardless of the initial geometry of the leg. Thus, we are confident that the method of numerical integration (4th order Runge Kutta) was appropriate for our study.

In our main analysis, we drove the forward model with the same profile of joint torques regardless of the initial geometry of the leg. The profiles we chose were *averages* of joint torques calculated in the inverse dynamic analysis for a small range of initial toe positions. This allowed us to examine how the rising slopes of toe trajectories were dependent on the initial geometry of the leg independent of the any changes in input commands. Our analysis was restricted to examining only leading steps of the right hind leg because the movement of the right leg (closest to the video camera) could be more accurately measured because the reflective markers were always visible, and the range on the initial toe distances from the barrier was much larger for the leading leg.

### Electromyographic (EMG) analysis of flexor muscle activity

In the three experimental animals, EMG recording electrodes were implanted in the two main knee flexor muscles, semitendinosus (St), and medial sartorius (Sart_*m*_), and the main hip flexor, iliopsoas (Ip) of the right hind leg. The procedures for implanting and recording EMGs have been fully described in previous papers (Donelan et al., 2009). The reason for recording flexor EMGs was to determine whether the magnitude of flexor burst activity during the early swing phase was dependent on initial leg geometry. If mechanical factors were entirely responsible for modifying the profiles of toe trajectories with different initial toe position, then we predicted that there would be little or no dependence of flexor EMGs magnitudes on the initial leg geometry.

The flexor EMG bursts were digitized using the analog acquisition hardware of the Peak Motus system, thus allowing synchronization of the video and EMG recordings. Data files of EMG activity stored by Peak Motus were then used in custom written Matlab programs to determine the magnitude of EMG bursts. The EMGs were full-wave rectified, low-pass filtered (cutoff = 50 Hz) and integrated over a time window of 150 ms from the beginning of the bursts.

## Results

### Forward modeling of toe trajectories

**Figure 3:**
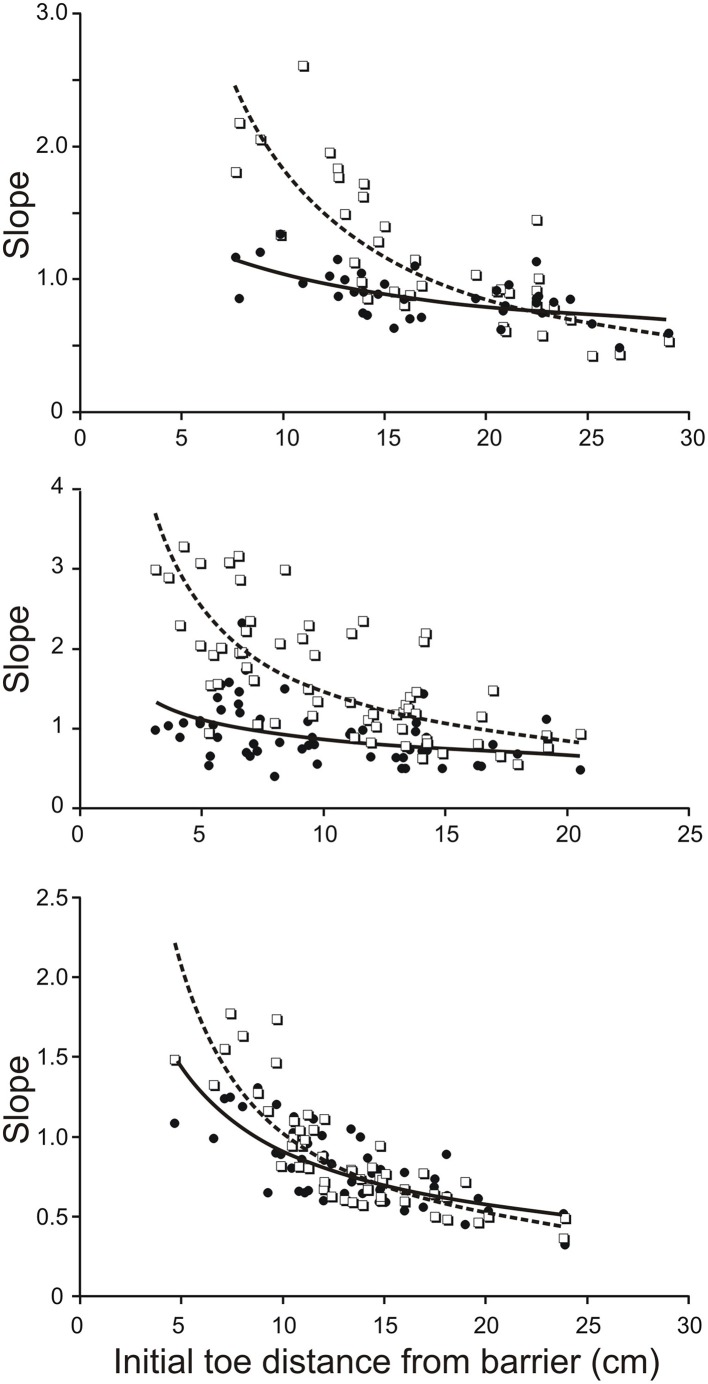
**Slope of toe trajectories increase with shorter initial toe distances from the barrier in the behaving animal (dash lines and open squares) and in the forward model (solid line and filled circles) driven by averaged torque profiles calculated for a subset of trials starting with toe-to-barrier distances in the range of 16–25 cms**. The initial geometry of the leg in each simulated trial was the same as initial geometry of the leg in the corresponding trial of the behaving animal. Note the divergence of the best best-fitting curves as the initial toe-to-barrier distance decreases due to the lower dependence of the modeled slopes on initial distance from the barrier. The three data sets are from three different animals.

To estimate the contribution of mechanical factors in increasing the slope of the trajectory of the toe when its initial position is close to the remembered barrier, we drove the four-link forward model of the hind leg with the *same* profile of torques for all initial positions of the hind leg. The torque profiles we chose were the averages of torque profiles for the subset of trials in which the initial toe position was at the longest distance from the barrier. Examples of torque profiles for the hip, knee, and ankle joints are shown in **Figure 5A**. Assuming that the initial mechanical arrangement of the leg does contribute to increasing the slope of the toe trajectory as the toe distance from the barrier decreases as described previously in behaving animals (Pearson and Gramlich, 2010), the main prediction of our forward modeling analysis was that the slopes of the toe trajectories produced by the simulation should increase as the initial toe distance from the barrier decreases. This prediction was found to be correct for the simulations based on biological data from three animals (Figure 3). Figure 3 shows scatter plots of the toe trajectory slopes vs. initial toe distances observed in the behaving animals (dotted lines, open squares) and produced by the simulation (solid line, filled circles). Both sets of data (biological and simulated) show the slope increasing as the toe-to-barrier distance decreases, but the rate of increase is greater for the behavioral data. The reasonably close correspondence between the observed and simulated slopes at large initial toe-to-barrier distances was expected because the simulation was driven by the average profiles of joint torques and angular velocities calculated for the subset of trials starting at large initial toe-to-barrier distances.

**Figure 4:**
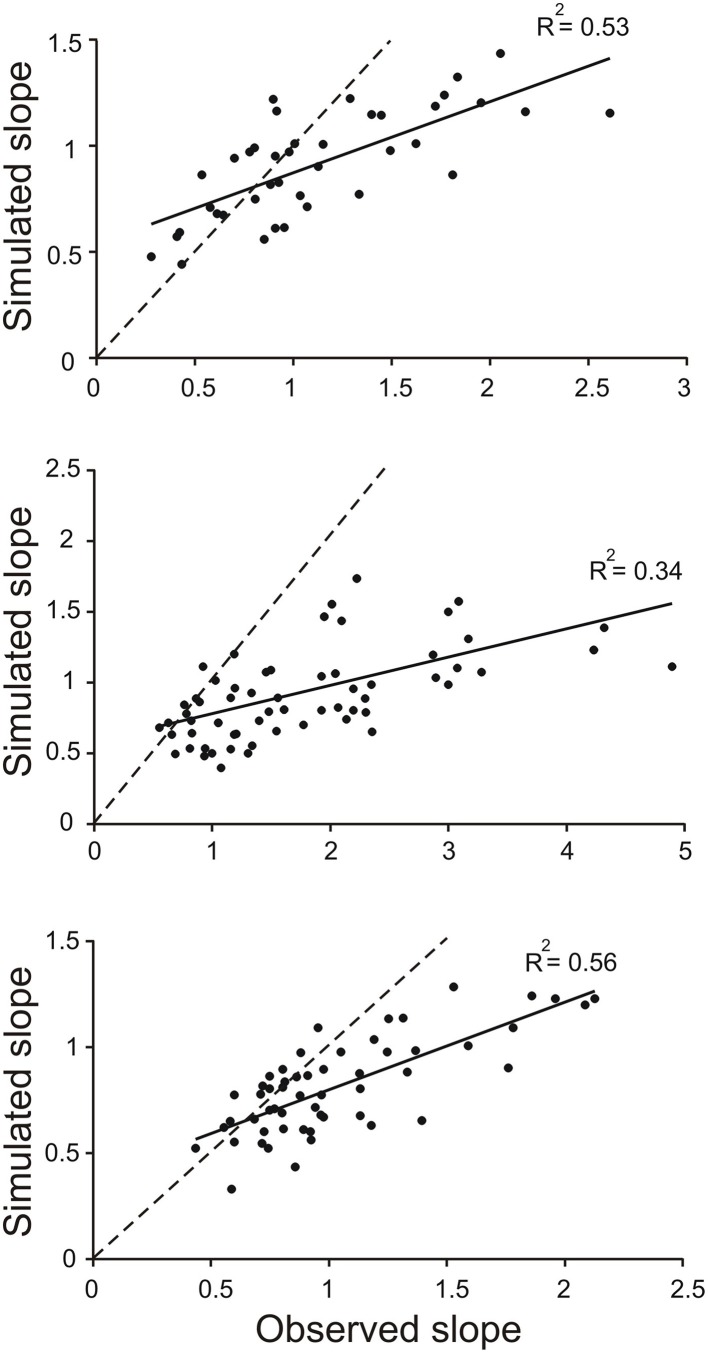
**Slopes of toe trajectories produced by the simulation are generally lower than the slopes for the observed toe trajectories**. Each data point in the scatter plots represents the slope of toe trajectory from the simulation (simulated slope) and the slope observed (observed slope) for a single trail. The three sets of data are from trials in three different animals. Note the divergence from the identity line (dashed) as slope increases. The matching of the data points at the low slopes is because the simulations were driven by average torque profiles of a subset of trials at long toe-to-barrier distances that are associated with low slopes (see Figure 3).

The divergence of the slopes observed in the simulations and in the behaving animals was clearly revealed in scatter plots of model-slope vs. actual-slope (Figure 4). The data points for all three animals are linearly related with the best fitting lines (solid lines) having slopes of 0.34, 0.20, and 0.41 (the dashed lines in Figure 4 are the identity lines with slopes of 1). This dependency was found to be significant in all three animals (*R*^2^ = 0.53, *p* < 0.001, *n* = 39; *R*^2^ = 0.34, *p* < 0.001, *n* = 55; *R*^2^ = 0.0.56, *p* < 0.001, *n* = 72; *T*-tested using TDIST in Microsoft Excel). Because larger slopes are associated with trials with short toe-to-barrier distance (Figure 3), the plots in Figure 4 show that the increase in model-slopes with shorter toe-to-barrier distance was less pronounced than the increase in actual-slopes with shorter toe-to-barrier distance. The matching of the model-slopes and actual-slopes at low slope values (close to the intersection of the solid and dashed lines) again reflects the fact that the simulation was driven by torque profiles associated with trials starting with large initial toe-to-barrier distance.

The slope increase in the simulated trajectories with decreasing toe-to-barrier distance indicates that mechanical factors related to leg geometry can significantly contribute to enhancing the elevation of the leg when starting a step close to the remembered barrier. But in addition, the divergence of the slopes of the modeled and actual toe trajectories indicates that neuronal commands to leg flexor muscles increases as the toe-to-barrier distance decreases. We predicted, therefore, that the flexion torque at one or more of the leg joints would increase as the toe-to-barrier distance decreased.

### Inverse dynamic analysis of hind leg stepping over a remembered barrier

**Figure 5:**
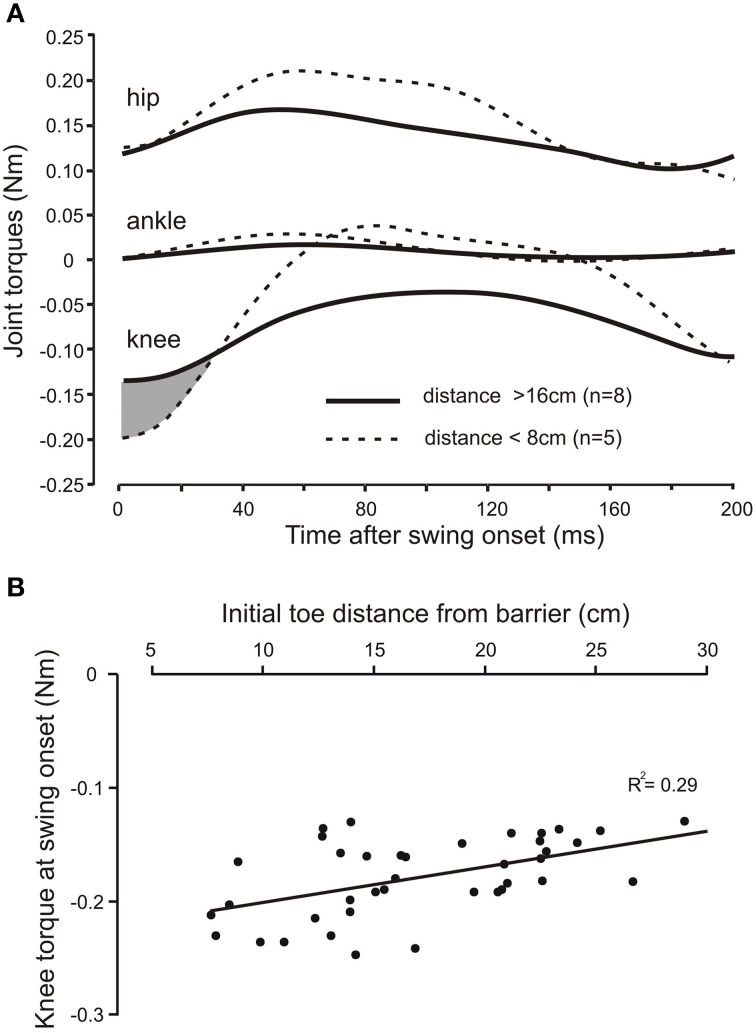
**The knee torque at the beginning of swing increases with shorter initial toe distance from the barrier**. **(A)** Averaged torque profiles hip, knee, and ankle joints for the first 200 ms of the swing phase for long (solid lines) and short (dashed lines) initial toe-to-barrier positions. Note the knee torque is larger for shorter distances for approximately the first 30 ms of swing (shaded area), whereas the initial torques at the ankle and hip joints are very similar at swing onset for both conditions. Knee flexion torques are negative for the geometry of the leg used in the inverse dynamic analysis, while hip and ankle flexion torques are positive. **(B)** Scatter plot of data from another animal showing the increase in the knee torque at swing onset with decreasing toe-to-barrier distance.

In the three adult cats we examined the relationship between the profiles of joint torques, derived from an inverse dynamic analysis of a leading hind leg stepping over the virtual barrier. The joint torque profiles were similar in all three animals. Figure 5A shows, for one animal, the average torque profiles at the hip, knee, and ankle joints for eight trials when the initial toe-to-barrier distance was greater than 16 cm (solid lines), and five trials when this distance was less than 8 cms (dashed lines). At all three joints there was an initial torque in the flexion direction (this is plotted in the negative direction for the knee because, by convention, torques are measured in the anticlockwise direction so the clockwise flexion torque at the knee in the leg geometry we analyzed (Figure 1) is negative. The most notable feature of the plots shown in Figure 5A is that for the hip and ankle joints the flexion torques at the start of swing are similar for the two starting positions, whereas the flexion torque at swing onset at the knee is larger for the steps starting close to the barrier than for the steps starting a long distance from the barrier. Figure 5B is a scatter plot of data from another animal showing a progressive increase in knee torque as the toe-to-barrier distance decreases. This dependency was found to be significant in all three animals (*R*^2^ = 0.290, *p* < 0.001, *n* = 39; *R*^2^ = 0.208, *p* < 0.001, *n* = 55; *R*^2^ = 0.121, *p* = 0.003, *n* = 72; *T*-tested using TDIST in Microsoft Excel). By contrast, there was no significant dependence of the initial torques at the hip and ankle joints on the initial distance of the toe from the barrier. *R*^2^ values for the hip joint were 0.009 (*p* = 0.82, *n* = 39), 0.001(*p* = 0.49, *n* = 55), and 0.003 (*p* = 0.63, *n* = 72) and for ankle they were 0.042 (*p* = 0.21, *n* = 55), 0.043 (*p* = 0.14, *n* = 55), and 0.002 (*p* = 0.71, *n* = 72). The dependence of knee torque on distance indicates that increases in neural commands to knee flexors is required to fully produce the appropriate amount of knee flexion to avoid the remembered barrier when the initial toe position is close to the barrier. That is, increased motor commands to knee flexor muscles are required to produce the needed increase in the initial slope of the toe trajectory.

### EMG analysis of activity in leg flexor muscles

**Figure 6:**
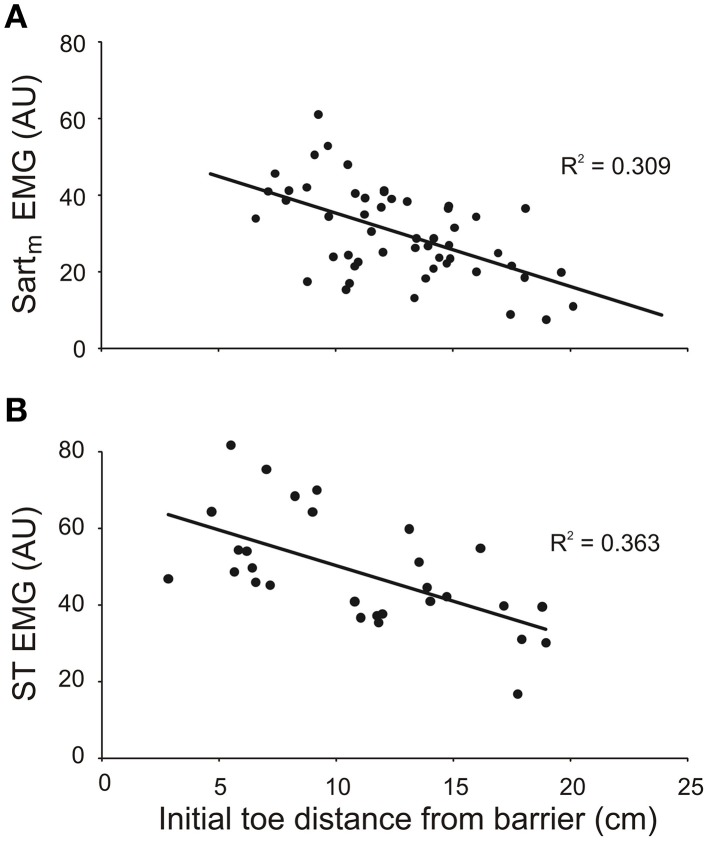
**The magnitude of EMG activity in knee flexor muscles semitendinosus (ST) and medial sartorius (Sart_*m*_) increases with shorter initial toe distance from the barrier**. The magnitudes of the EMGs were measured over the first 150 ms of burst onset. Data in **(A)** and **(B)** are from two different animals. EMG amplitudes measured in arbitrary units (AU).

The results from both the inverse dynamic analysis and the examination of the properties of the forward model indicated that changes in neuronal commands, in addition to changes in leg geometry, are involved in establishing the inverse relationship between the slope of toe trajectories and the initial toe-to-barrier distance (Figure 3). Thus, we predicted that the magnitude of bursts of activity in one or more leg flexor muscles would depend on the initial toe-to-barrier distance. We focused primarily on the activity in knee flexor muscles (ST and Sart_m_) because the inverse dynamic analysis showed that the knee flexion torque increased as the toe-to-barrier distance decreased (Figure 5). Consistent with this observation we found that the magnitude of the initial burst activity in both ST in all three animals and Sart_m_ in two animals (recording electrodes in the third malfunctioned) increased the closer the initial toe position was to the barrier (Figure 6). This dependency was found to be significant in all cases (*p* < 0.001; *T*-tested using TDIST in Microsoft Excel). EMG recordings from the hip flexor IP muscle in two animals did not reveal any dependence on the initial toe-to-barrier distance (*p* > 0.1 in both animals), which was consistent with the absence of any dependency of the initial hip torque on distance (see previous section).

## Discussion

A primary goal of the current investigation was to determine the extent to which purely mechanical factors might contribute to the modification of the toe trajectories when cats step over a remembered barrier with their hind legs. This investigation was motivated, in part, by an earlier finding that if a hind leg changes its initial position relative to a remembered barrier then the toe trajectory is appropriate for the new position and not the position at the moment the animals stopped walking (Pearson and Gramlich, 2010). This observation was interpreted as supporting the hypothesis that the neuronal system representing the memory of the position of the barrier with respect to the body could be updated to take into account the new position of the leg. However, an alternative possibility not considered in our earlier study, is that the modification of toe trajectory is entirely the result of changes in the initial geometry of the leg. In other words, updating the memory system regulating stepping over the remembered barrier is not required to generate a change in toe trajectory when a postural adjustment is made prior to stepping over the remembered barrier.

The results of the current investigation revealed that mechanical factors alone can contribute to altering toe trajectory in the correct direction but, in addition, changes in neuronal command to knee flexor muscles are required to fully produce the appropriate initial toe trajectory. The evidence for a contribution of mechanical factors came from the analysis of toe trajectories generated by a four-link forward dynamic model of the hind leg. By keeping the torque profiles at the four joints the same for all initial geometries of the leg, we found that the slope of the toe trajectories in the simulations increased as the toe was placed closer to the barrier (Figure 3). This is most likely related to the fact that when stepping began with the toe close to remembered barrier the leg was initially more flexed and the gravity torque at the knee was lower. Thus, this would have enabled the same motor commands (represented by joint torques) to more effectively elevate the leg. However, this mechanical contribution to the enhancement of toe elevation cannot account entirely for the toe elevations observed in behaving animals. This conclusion follows from our finding that mechanical factors alone are *not* sufficient to produce the required increase in the slope of toe trajectories as the toe-to barrier distance decreases (Figures 3, 4). The difference in the slopes of toe trajectories seen behaviorally and those produced by the simulation diverged as the initial toe position moved closer to the barrier. This divergence indicated that increased motor commands to leg flexor muscles must also have contributed to producing the steeper toe trajectories the closer the initial toe position was to the barrier.

Evidence for an increasing contribution of motor commands to leg flexor muscles when the initial toe position was closer to the barrier came from both the inverse dynamic analysis and the EMG recordings from leg flexor muscles. As the initial toe-to-barrier distance decreased, the initial flexion torque at the knee increased (Figure 5), whereas initial flexion torques at the hip and ankle joints remained almost constant. This finding indicates that the primary neuronal mechanism contributing to the regulation of toe trajectory is alterations in the level of motor commands to knee flexor muscles. Consistent with this conclusion was our finding that the magnitude of EMG bursts in both ST and Sart_m_ were larger the closer the initial toe position was to the barrier (Figure 6). Because the motor commands for producing the stepping movements depend critically on information about the barrier height stored in a long-lasting working memory (Pearson and Gramlich, 2010), our finding of the modulation of magnitude of the EMG bursts in knee flexor muscles strongly indicates that information about initial toe-to-barrier distance is also stored in this working memory system. Moreover, the fact that increased knee flexor muscle activation is required the closer the toe is to the barrier, and because the kinematics of toe trajectories for steps with and without prior postural adjustments are similar (Pearson and Gramlich, 2010), it seems very likely that information about toe position stored in the working memory system can be updated if the position of the toe is changed prior to stepping.

The general conclusion of this investigation is that both mechanical and neuronal mechanisms contribute to establishing the kinematic profile of toe movement when a leading hind leg of a cat steps over a remembered barrier. It is not possible to accurately estimate the relative contribution of these two mechanisms because the computational analysis was carried out using a highly simplified model of the hind leg. For example, we used joint torques as a proxy for neural commands thus ignoring the role of muscle properties and muscle moment arms in the transformation of neural commands to joint torques. Moreover, no consideration was given to the influence of viscoelastic properties of muscle and connective tissue. Nevertheless, our model was clearly sufficient to demonstrate qualitatively that variations in the geometry of the leg can influence the kinematics of toe trajectories in a manner that is appropriate for partially explaining the variation in toe trajectories in behaving animals.

### Conflict of interest statement

The authors declare that the research was conducted in the absence of any commercial or financial relationships that could be construed as a potential conflict of interest.
